# Sensory Neuron Development in Mouse Coccygeal Vertebrae and Its Relationship to Tail Biopsies for Genotyping

**DOI:** 10.1371/journal.pone.0088158

**Published:** 2014-02-04

**Authors:** Jerald Silverman, Gregory Hendricks

**Affiliations:** 1 Department of Animal Medicine, University of Massachusetts Medical School, Worcester, Massachusetts, United States of America; 2 Department of Cell Biology, University of Massachusetts Medical School, Worcester, Massachusetts, United States of America; Tokyo Medical and Dental University, Japan

## Abstract

A common method of genotyping mice is via tissue obtained from tail biopsies. However, there is no available information on the temporal development of sensory neurons in the tail and how their presence or absence might affect the age for performing tail biopsies. The goals of this study were to determine if afferent sensory neurons, and in particular nociceptive neurons, are present in the coccygeal vertebrae at or near the time of birth and if not, when they first can be visualized on or in those vertebrae. Using toluidine blue neuronal staining, transmission electron microscopy, and calcitonin-related gene peptide immunostaining, we found proximal to distal maturation of coccygeal nerve growth in the C57BL/6J mouse. Single nerve bundles were first seen on postpartum day (PPD) 0. On PPD 3 presumptive nociceptive sensory nerve fibers were seen entering the vertebral perichondrium. Neural development continued through the last time point (PPD 7) but at no time were neural fibers seen entering the body of the vertebrae. The effect of age on the development of pain perception in the neonatal mouse is discussed.

## Introduction

One of the most common forms of genotyping mice is via tissue obtained from tail biopsy (tailing) [1], [2]. However, there is little if any information as to whether the procedure might damage nerves associated with the biopsied areas. More than 150 years ago, researchers identified sensory nerve fibers on and within the long bones of rodents (reviewed in reference [3]). These sensory fibers are essential for an animal’s responsiveness to noxious stimuli affecting the bone and periosteum, but also may serve an important role in regulating blood flow and erythrogenesis within the marrow [4] and stimulating osteoblasts and inhibiting osteoclasts [5]–[7]. In contrast to the long bones, the irregularly shaped coccygeal vertebrae have not been extensively researched relative to the development of their innervation. Perhaps this is because they are no more than “miniature long bones” [8], an observation supported, in part, by the fact that the osseous development of the coccygeal vertebrae is similar to that of long bones [9], [10]. Nevertheless, the literature on the coccygeal vertebrae of mice and rats is not entirely barren. For example, the morphology of mouse coccygeal vertebrae has been described [11] as has their general growth and ossification pattern [9], [12]. The effects of hypoxia on mouse coccygeal vertebral development [8] and the development of vasomotor innervation in the rat tail [13] also have been studied. But quite unlike long bones, there are no studies addressing the early stages of sensory nerve growth on and within the coccygeal vertebrae of the laboratory mouse.

DNA for genotyping mice can be isolated from tissues such as the animals’ tail, ear, blood, or hair. As noted earlier, cutting off a small piece of tail, typically 3–5 mm from animals less than 1 week to more than 4 weeks of age [14], is often the preferred method for obtaining tissue for genotyping; however, that procedure injures all tissues in the path of the cutting blade, including sensory neurons, if present. The degree of pain an animal experiences from tailing is likely to be a function of the tissues injured, the maturation of the animal’s peripheral and central nervous systems, and the presence or absence of nociceptive neurons at or near the site of injury. Observations of mice on their day of birth readily demonstrate that these neonates are capable of moving their tails, thereby indicating the presence of functional motor neurons and muscles in the tail. Most likely, this movement is little more than uncontrolled spontaneous twitching. It is not known though, if afferent sensory neurons, and in particular nociceptive neurons, are present in the coccygeal vertebrae at or near the time of birth (as they are with long bones) and if not, when they first can be visualized in those vertebrae. We have answered these questions and extrapolated our findings to their possible impact on pain from tailing.

## Materials and Methods

### Ethics Statement

This study was performed in accordance with the recommendations of the *Guide for the Care and Use of Laboratory Animals* of the U.S. Institute for Laboratory Animal Research, National Research Council [15] and carried out under approval A-2301-11 from the Institutional Animal Care and Use Committee of the University of Massachusetts Medical School.

### Animals

One timed-pregnant C57BL/6J mouse (The Jackson Laboratory, Bar Harbor, ME) gave birth to 9 pups on postpartum day (PPD) 0. On that day, 3 of the pups were briefly taken from their dam and had the distal 5 mm of their tails removed by a quick cut with a new razor blade. They were then wiped with bedding from their home cage and uneventfully returned to their dam. On PPD 3 the same procedure was performed on 3 different mice from the same litter and on PPD 7 the same procedure was performed on the remaining 3 pups. Externally, the tail of a newborn C57BL/6J mouse is about 1.25 cm in length. We removed 5 mm of the tail as it was the largest section that we were confident could easily and safely be removed from a neonatal mouse [16] and it would allow us to readily integrate our findings with those of a previous study which evaluated the temporal development of mouse coccygeal vertebrae [12].

**Figure 1:**
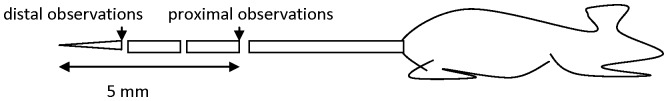
Locations of tail biopsy examination sites. A 5/6 mice at 0, 3, and 7 days postpartum (PPD). The tissue was cut into 3 pieces and prepared for toluidine blue staining, transmission electron microscopy (TEM), and calcitonin gene-related peptide (CGRP) immunostaining. The arrows indicate the areas examined by TEM and bright field microscopy.

Each tail tip was cut into 3 approximately equal sections of just under 2 mm in a manner that allowed for the identification of the proximal and distal ends of each section. The middle section shown in Figure 1 was not used in this study but was cut to allow for improved fixation of the proximal and distal segments.

Tissue used for immunohistochemistry to detect calcitonin gene-related peptide (CGRP) was obtained from six C57BL/6J pups born to a second dam. On PPD 0, 3 and 7, two different pups (at each time point) had the distal 5 mm of their tails biopsied as described above.

### Epoxy Resin Embedding for Transmission Electron Microscopy (TEM)

Tail segments were fixed by immersion in 2.5% (v/v) glutaraldehyde in 0.5 M Na phosphate buffer (pH 7.2) for 1 hour at room temperature. The fixed samples were washed 3 times in the same buffer. Following the third wash the tail segments were post-fixed for 1 hour in 1% osmium tetroxide (w/v) in the same buffer, washed 3 more times and left overnight at 4°C in fresh buffer. The next morning the samples were dehydrated through a graded series of ethanol to 100% and then transferred into propylene oxide (2 changes) and finally into epoxy resin/propylene oxide and left overnight to complete infiltration. Following infiltration the tissues were transferred through 3 changes of fresh epoxy resin and embedded in flat molds and polymerized at 70°C. The polymerized blocks were then oriented so as to cut cross-sections of the tail segments. One micron sections were taken and stained with 1% toluidine blue and examined by light microscopy for histological appearance and to select areas for correlative TEM. Electron micrographs were collected using a Philips CM 10 transmission electron microscope (Koninklijke Philips, Amsterdam, Netherlands) equipped with a Gatan, Erlangshen 785 digital camera system (Gatan, Pleasanton, CA).

### Acrylic Resin Embedding for Immunostaining CGRP

Tail segments were fixed by immersion in 4% paraformaldehyde for 1 hour at room temperature. The segments were then washed overnight in 0.5 M Na phosphate buffer (pH 7.2). Following the overnight wash the tail segments were dehydrated through a graded series of ethanol to 100% (2 changes) and then transferred into a 50∶50 (V/V) mixture of LR White acrylic resin and 100% ethanol and left overnight to infiltrate. The following morning the tail segments were transferred through 3 changes of fresh LR White acrylic resin (1 hour each) and then transferred into Beem® capsules, capped, and placed in a 70°C oven to polymerize. One micron sections were cut, stained with 1% toluidine blue, and examined by light microscopy for histological appearance. Consecutive sections were incubated with rabbit anti-CGRP (Sigma-Aldrich #C 8198, St. Louis, MO) for 4 hours at room temperature, washed and incubated with Texas Red®-X goat anti-rabbit IgG (Life Technologies #T6391, Grand Island, NY) in the dark for 1 hour at room temperature. Images were recorded using an Olympus AX90 microscope with an automated stage (Olympus America, Melville, NY) and a Q-Color5 5MP digital camera system (Olympus America, Melville, NY).

## Results

### PPD 0

**Figure 2:**
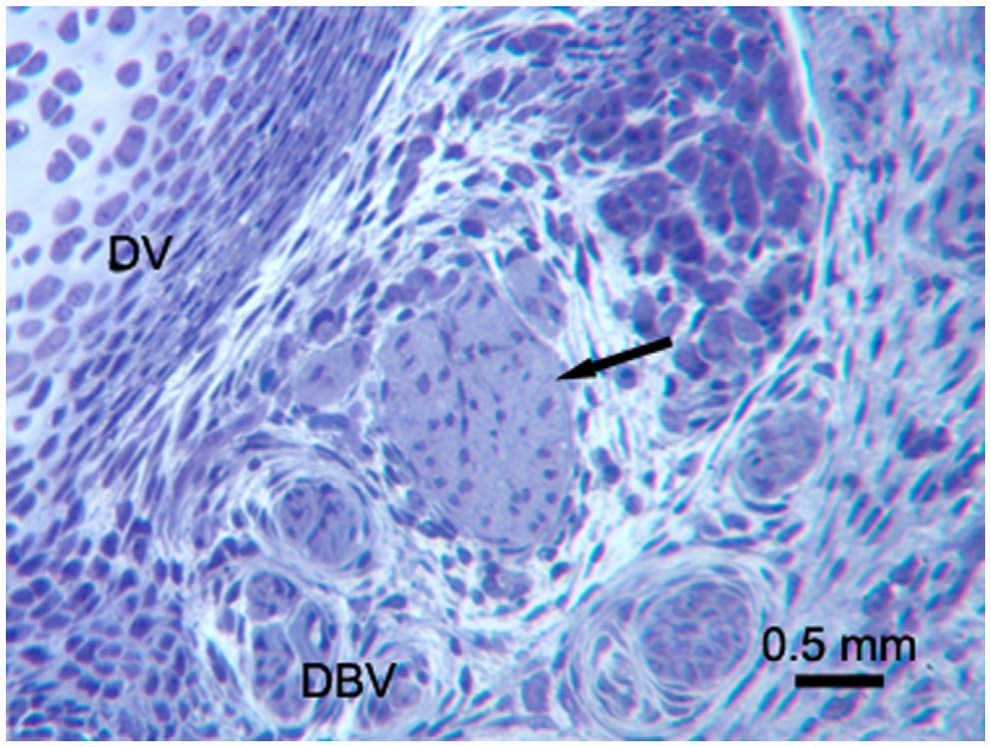
PPD 0, proximal biopsy site. Cross section with toluidine blue staining of a 1 µm epoxy section. An immature nerve bundle (arrow) in the center of the image is clearly visible. DV (developing vertebra), DBV (developing blood vessel).

**Figure 3:**
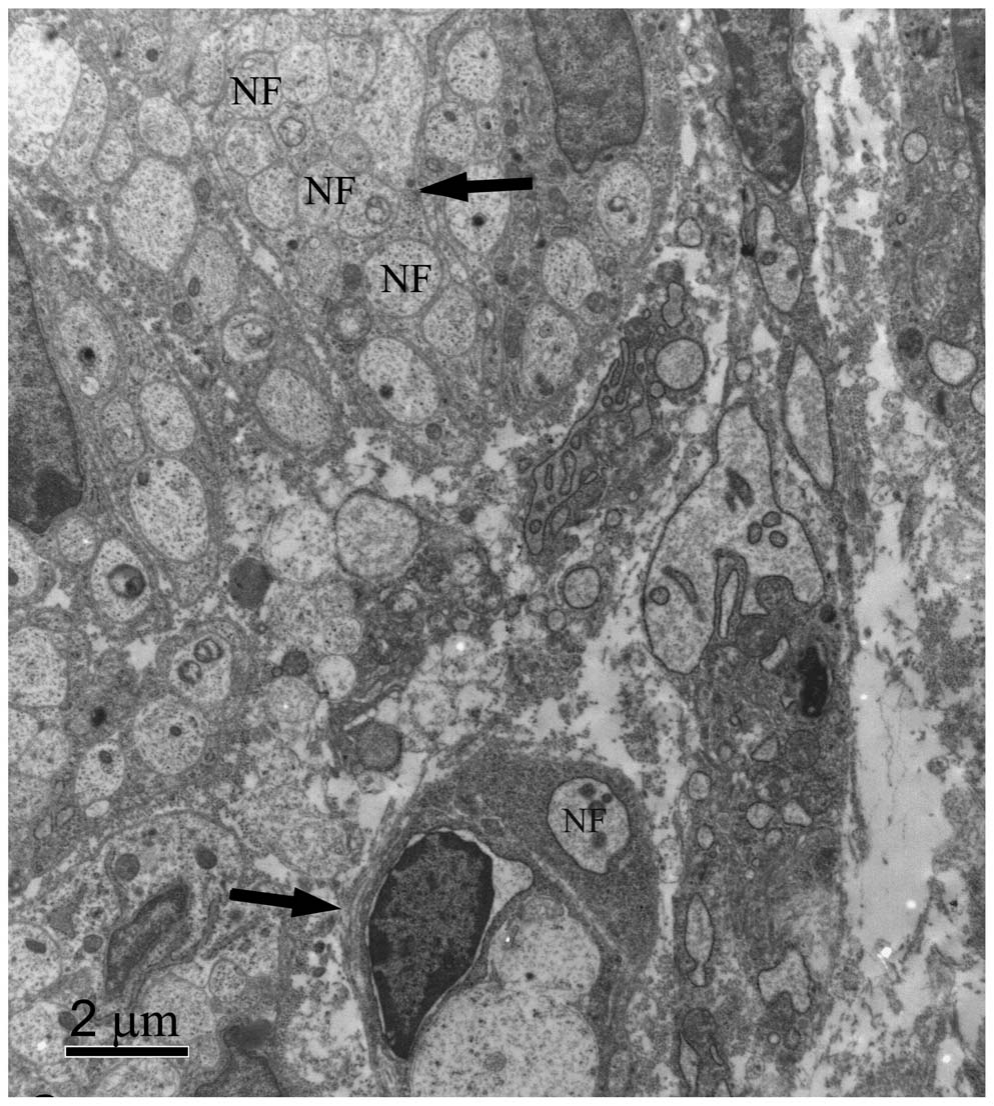
PPD 0, proximal biopsy site, TEM. This thin cross-section shows that individual nerve fibers (NF) within the nerve bundle (upper arrow) on the micrograph appear unmyelinated and no Schwann cells are present. Note the small blood vessel lower center (lower arrow), with what appears to be a small unmyelinated nerve fiber (NF) running next to it surrounded by extracellular matrix.

**Figure 4:**
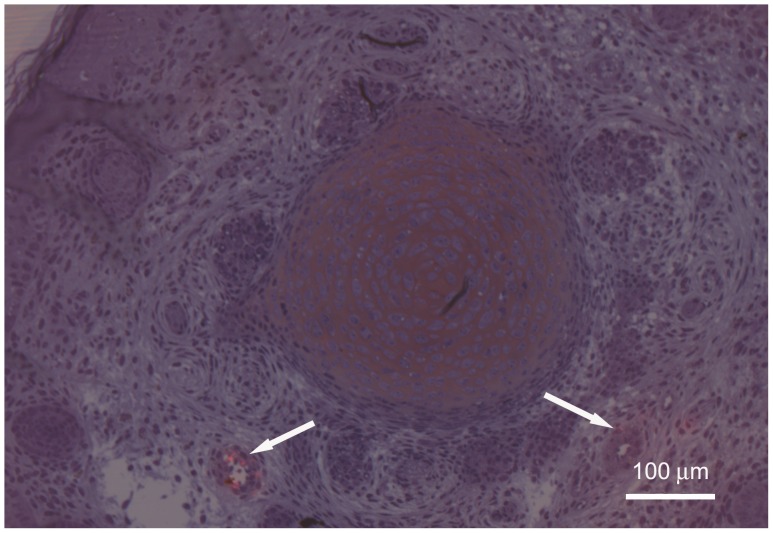
PPD 0. Proximal biopsy site. Adjacent 1 µm LR White acrylic resin sections were merged to show both the Texas Red fluorescent image for CGRP staining and the tissue structure seen with toluidine blue staining. Note that the only evidence of CGRP neural staining is around the small blood vessels (arrows) lower left and right. DV denotes the developing vertebra in the center of the image. The diffuse reddish color is most likely CGRP staining of osteoclasts and possibly osteoblasts, resulting from the early mineralization of the vertebra.

Toluidine blue staining on PPD 0 demonstrated single, discrete nerve bundles at the proximal biopsy site (Figure 2). The nerve bundles appeared small and unmyelinated. These results were confirmed by TEM (Figure 3). Toluidine blue staining and TEM of the distal biopsy site displayed a general lack of organization of the developing tissues. CGRP immunostaining indicated that at this age the putative sensory nerves were only along blood vessels, not developing bone (Figure 4).

### PPD 3

**Figure 5:**
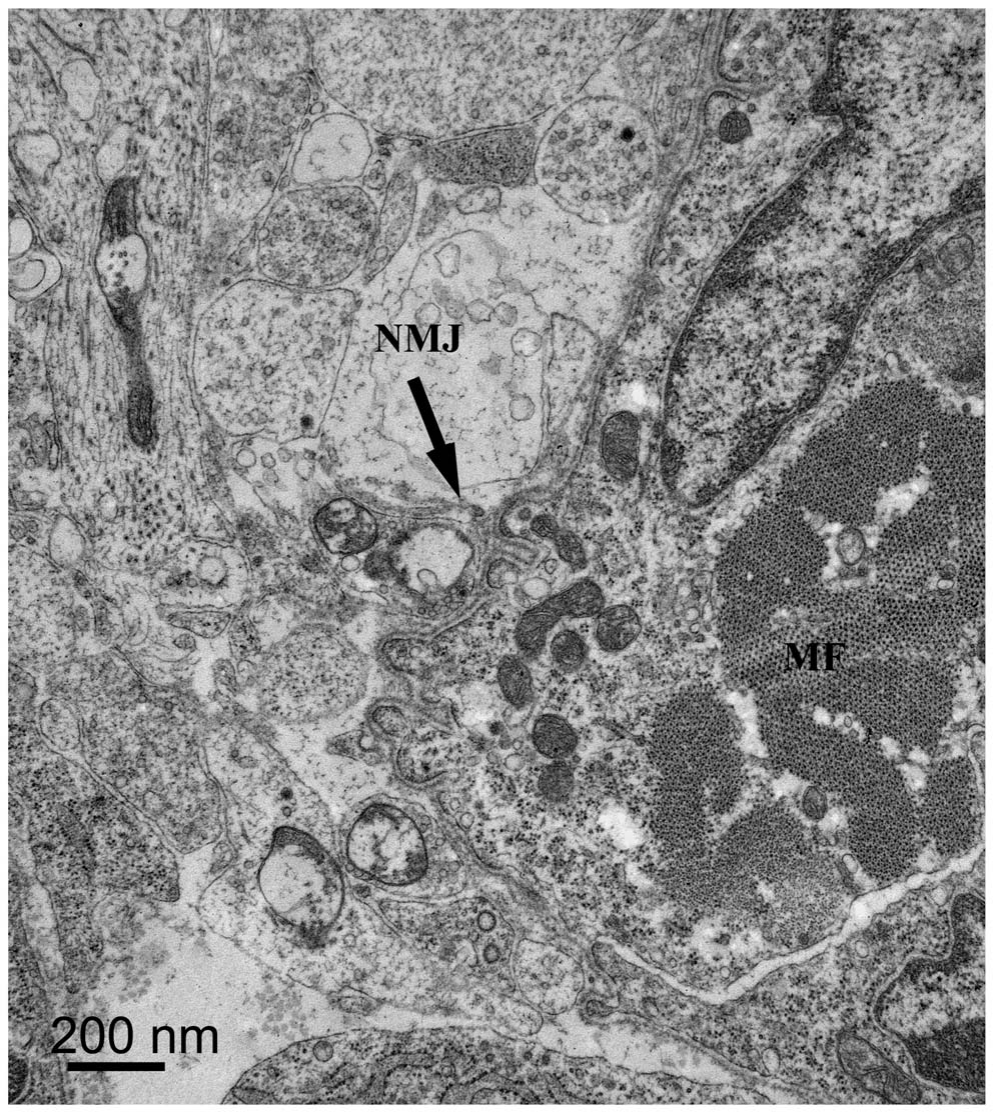
PPD 3, proximal biopsy site, TEM. This thin cross-section shows a neuro-muscular junction (NMJ) below the arrow center of image, on a small muscle cell cross-section (MF).

**Figure 6:**
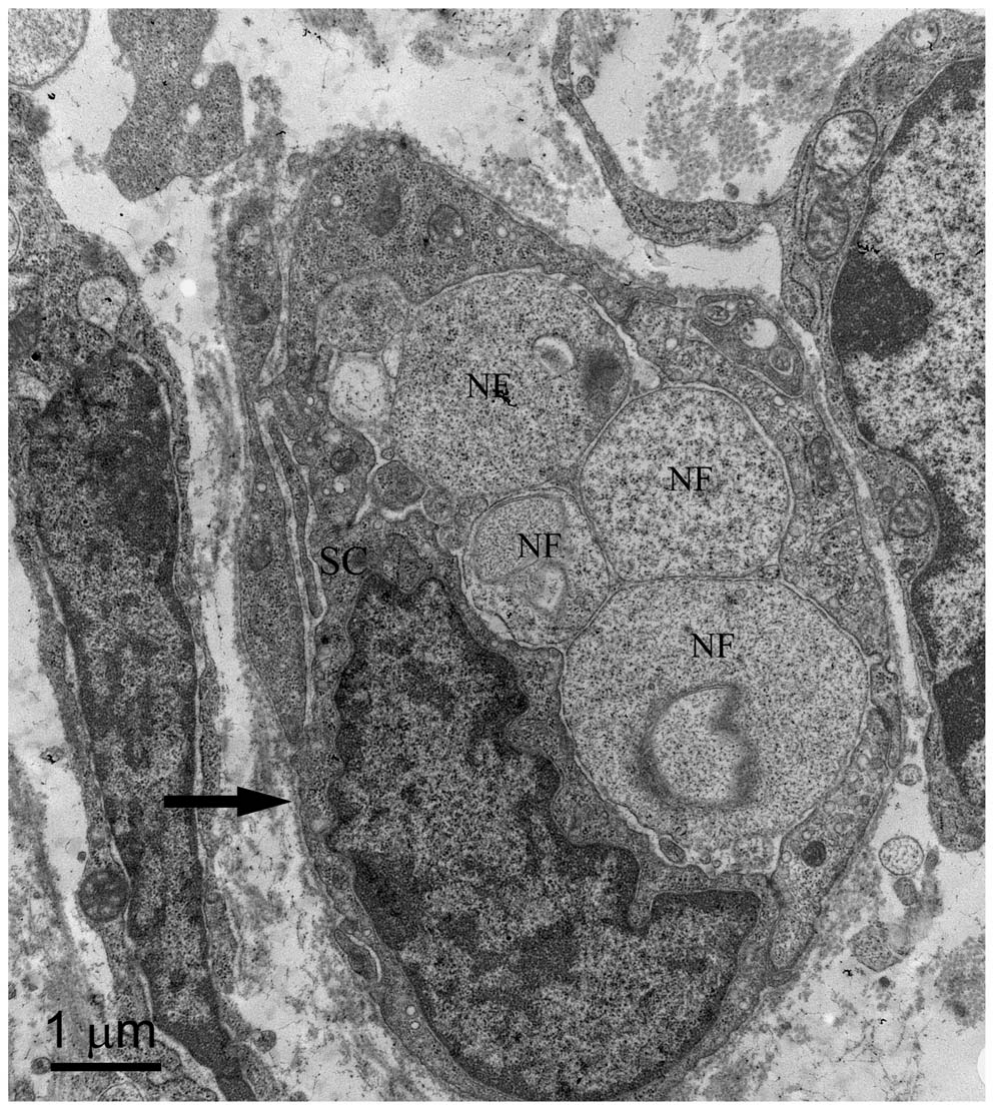
PPD 3, proximal biopsy site, TEM. A thin cross-section shows a nerve fiber in the periosteum with multiple nerve fibers (NF) surrounded by a single Schwann cell (arrow points to the dark nucleus of the Schwann cell).

**Figure 7:**
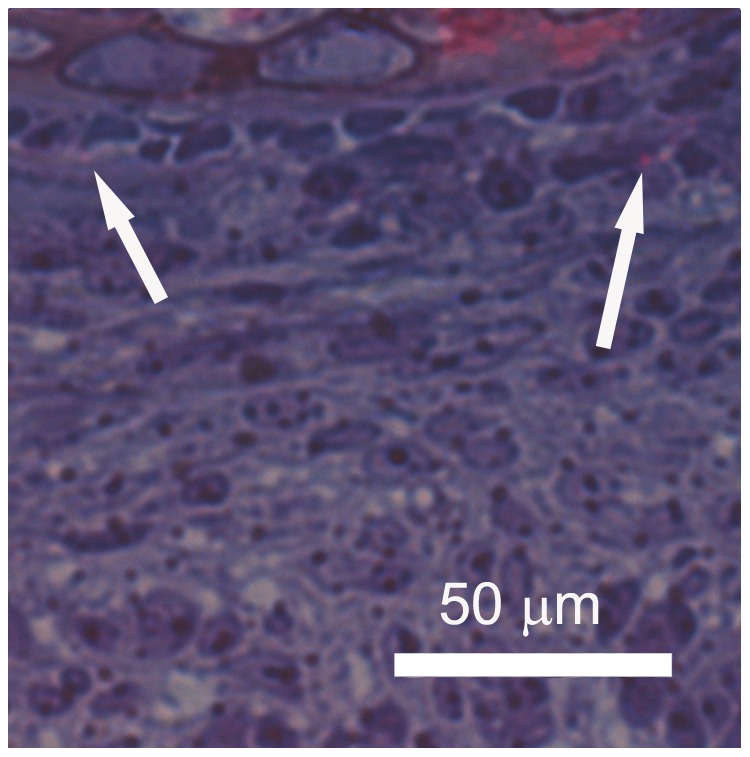
PPD 3, proximal biopsy site. Adjacent 1 µm LR White acrylic resin sections were merged to show both the Texas Red fluorescent image for CGRP staining and the tissue structure seen with toluidine blue staining. Arrows indicate the first faint evidence of CGRP staining in the periosteum. The structure at the top of the image is the developing vertebra and the diffuse reddish color is most likely CGRP staining of osteoclasts and possibly osteoblasts, resulting from the early mineralization of the vertebra.

Toluidine blue staining indicated nerve bundle organization at the proximal biopsy site. TEM at the proximal site revealed a neuromuscular junction in one of the muscle bundles (Figure 5) and numerous nerve fibers going to and through the forming periosteum (Figure 6). None of the fibers seen were myelinated but some had Schwann cells associated with them. The nerves were still single, discrete bundles. At the proximal site CGRP staining revealed immunoreactivity around blood vessels and what appeared to be the first evidence of putative sensory nerve fibers in the periosteum (Figure 7). There also was generalized CGRP staining of the perichondrium, most probably due to the affinity of immunoreactive CGRP with CGRP receptors in osteoclasts and osteoblasts of the mineralizing vertebra [7], [17], [18]. At the distal biopsy site, CGRP immunostaining of nerve fibers still was present only around blood vessels.

### PPD 7

**Figure 8:**
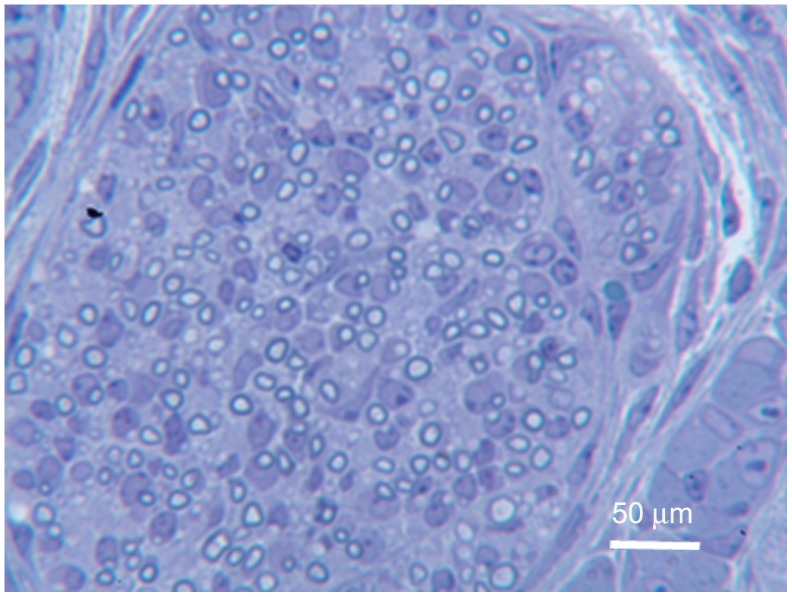
PPD 7, proximal biopsy site, toluidine blue stain. This cross-section shows that many of the nerve fibers are now myelinated (dark rings) and increased in number.

**Figure 9:**
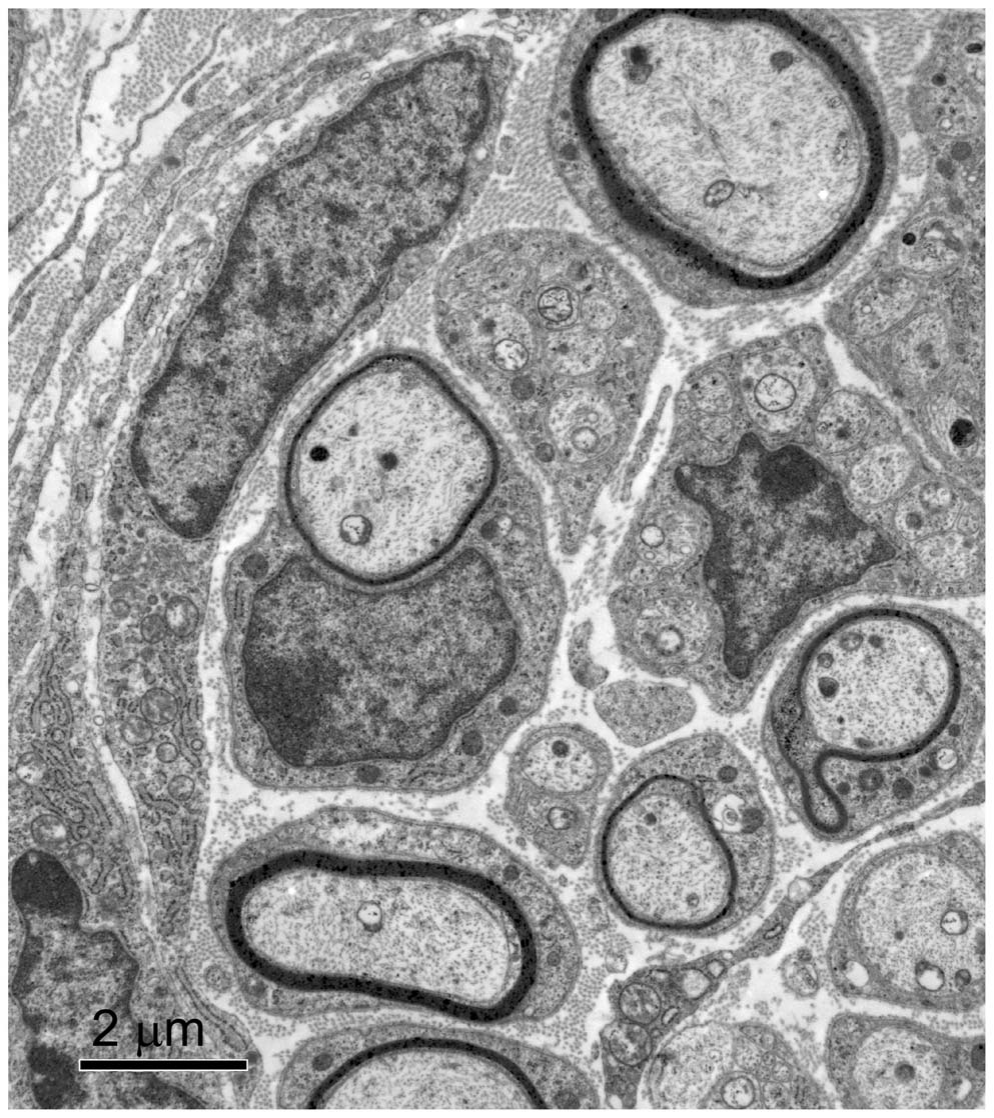
PPD 7, proximal biopsy site, TEM. In this thin cross-section there is a nerve bundle with numerous fiber tracks that are now myelinated to some extent and surrounded by a Schwann cell as indicated by the thick dark staining ring around some cells.

Toluidine blue staining at the proximal biopsy cut showed that the tissues were well organized, the nerve bundles appeared larger than on previous days, and the bundles contained both myelinated and unmyelinated axons that were found alongside blood vessels and in the developing periosteum. Myelinated individual nerve fibers were readily detected (Figure 8). The toluidine blue observations from the proximal biopsy site were confirmed by TEM examination (Figure 9).

**Figure 10:**
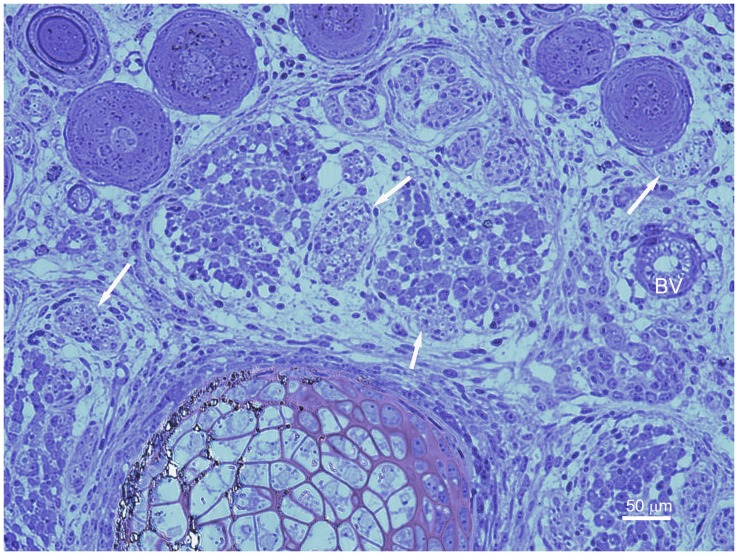
PPD 7, distal biopsy site, toluidine blue stain. In this cross-section the nerve has split into several distinct tracks (arrows).

**Figure 11:**
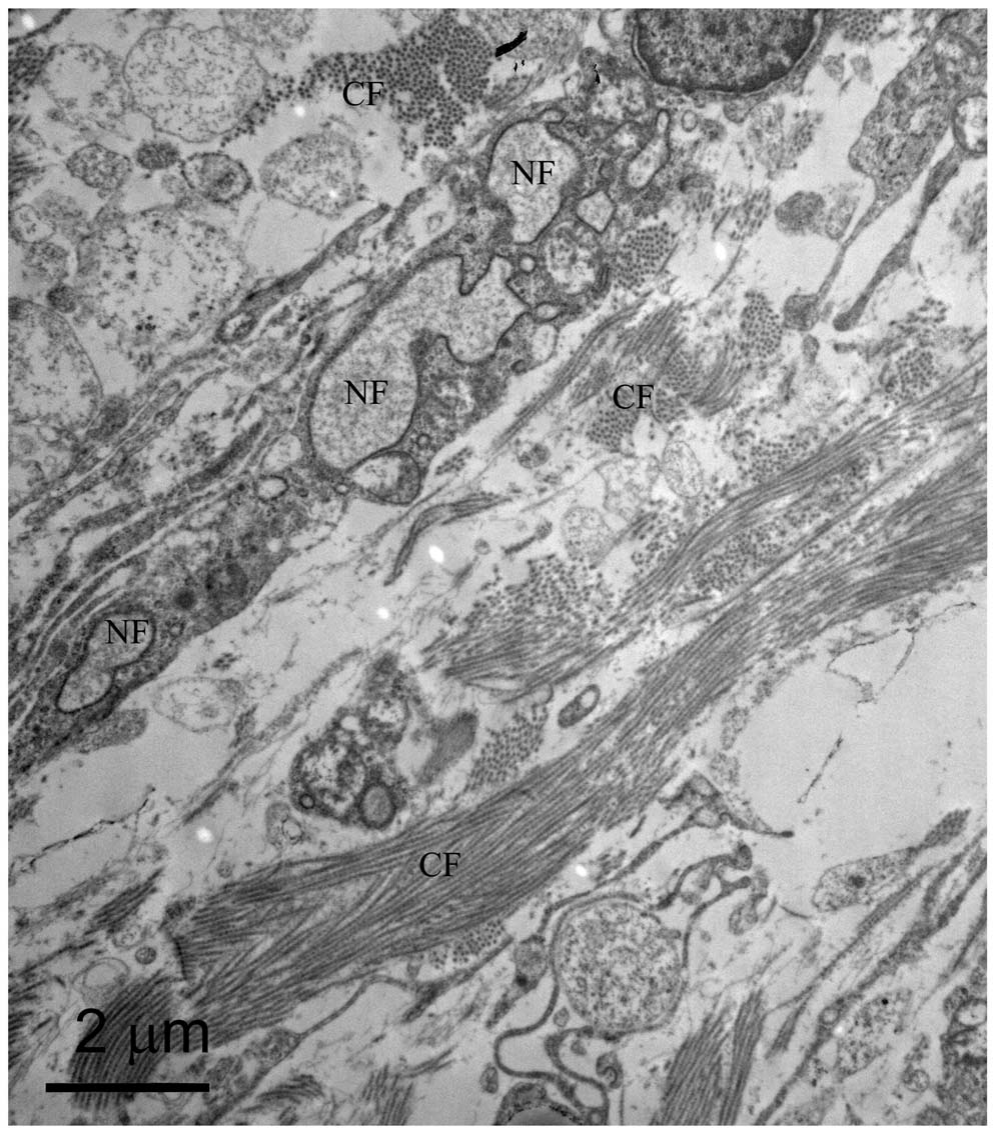
PPD 7, distal biopsy site, TEM. In this thin cross-section there is a nerve fiber (NF) in the periosteum surrounded by collagen filaments (CF).

At the distal biopsy site on PPD 7, toluidine blue staining showed that one or more nerves had split into several nerve bundles (not just a single bundle as found at PPD 0 and 3) and they were coursing alongside small blood vessels (Figure 10). With TEM we found nerve fibers in the forming periosteum (Figure 11) and unmyelinated axons were readily detected.

**Figure 12:**
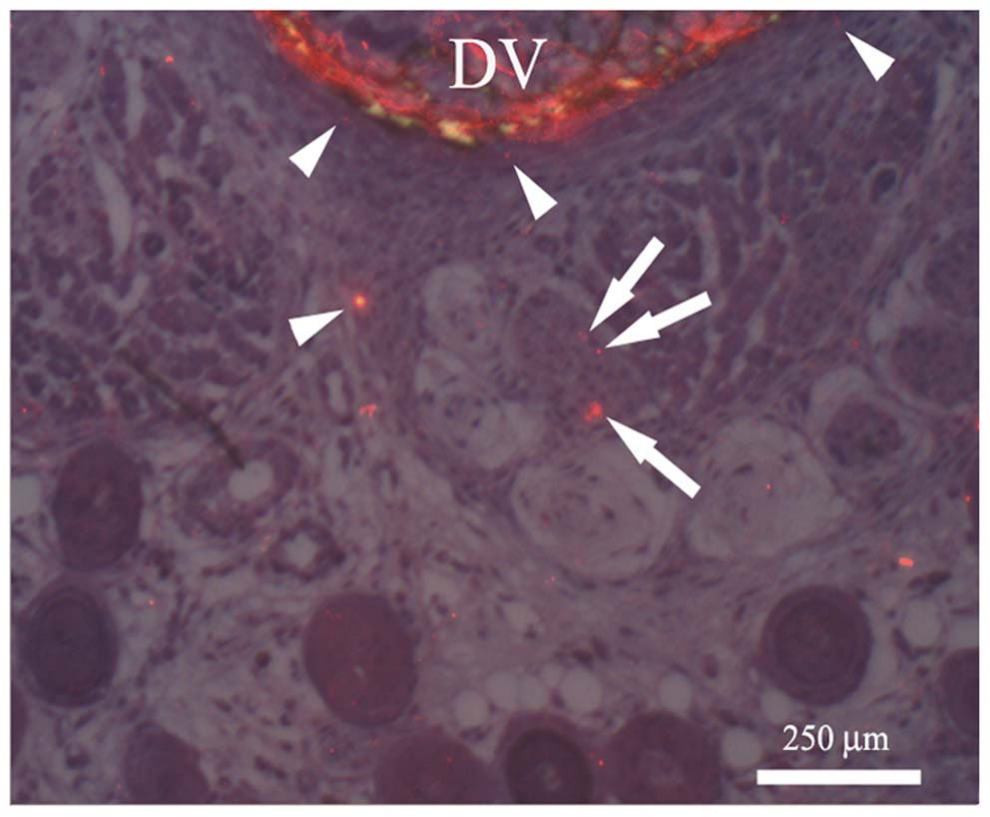
PPD 7, proximal biopsy site. Adjacent 1 µm LR White acrylic resin sections were merged to show both the Texas Red fluorescent image for CGRP staining and the tissue structure seen with toluidine blue staining. Arrows indicate the first evidence of CGRP staining in a nerve bundle. There is significant CGRP staining in the periosteum (arrowheads). The partial ring with diffuse reddish color in the developing vertebra (DV) is most likely CGRP staining of osteoclasts and possibly osteoblasts, resulting from the early mineralization of the vertebra.

At the proximal biopsy site CGRP immunostaining revealed reactivity around blood vessels and what appears to be the first evidence of a sensory nerve fiber staining in a nerve bundle, along with the immunostaining of the forming periosteum (Figure 12) which we first found on PPD 3. CGRP immunostaining at the distal site was similar to the proximal site.

## Discussion

The coccygeal vertebrae of mice and rats increase in number and mature in a proximal to distal direction [9], [11], [12]. The present data demonstrate that the nerve supply to the coccygeal vertebrae of the C57BL/6J mouse also has proximal to distal maturation. This was anticipated as the sensory nerve supply to mouse and rat long bones exhibits proximal to distal growth and maturation [3], [7], [19]. Because the proximal biopsy site was about 0.5 cm distal to the base of the animals’ tail, it is likely that neural development of the vertebrae closer to the sacrum occurred slightly earlier than our findings from the proximal biopsy site that we used.

With toluidine blue staining, single nerve bundles were first seen on PPD 0 at the proximal biopsy site along with limited CGRP immunoreactivity. By PPD 3 there was continued growth of nerve bundles, nerve fibers were seen in the perichondrium, and there was early CGRP immunoreactivity of the perichondrium, primarily at the proximal biopsy site. By PPD 7 the neural development continued at both the proximal and distal biopsy sites. At the proximal site CGRP immunoreactivity was present in the perichondrium and individual nerves fibers were in nerve bundles; however, even at this age, we did not visualize nerves or nerve fibers entering the vertebral body although fibers may have entered the vertebral body in areas not sectioned for this study.

The neuropeptide CGRP is produced by peripheral and central nervous system neurons, including those of bony tissue. It has been found to participate in the innervation of almost all rat long bones and surrounding soft tissue [5], [20] and in rats it has been detected in the lumbar vertebral body [21] and the coccygeal intervertebral disc [22]. Because CGRP can function in the transmission of nociceptive signals, its detection by immunohistochemistry has frequently been used as a marker of the presence of nociceptive nerve fibers [7], [22]–[25].

Using CGRP and Substance P as markers, sensory nerve fibers were visualized in rat long bone periosteum prior to birth [24]. Most of these sensory nerves were located close to blood vessels [20]. They first entered long bone diaphyses and then reached the epiphyses by PPD 7–8 [7], [26]. In the present study CGRP immunostaining indicated that presumptive sensory fibers entered the perichondrium of the developing coccygeal vertebra by PPD 7, although weakly staining presumptive sensory fibers were found on and in the perichondrium as early as PPD 3. Myelinated nerve fibers first appear at PPD 14 in the rat femur [3], differing from our finding of 7 days in the proximal coccygeal vertebrae. Todd and Tokito [13], using TEM, identified *vasomotor* innervation of the proximal end of the tail of the rat at PPD 3 but the nerve fibers did not have a Schwann cell covering (i.e., there was no early myelination). In the present study we also found at PPD 3 (using TEM) unmyelinated nerve bundles running alongside coccygeal blood vessels and at PPD 7 we found myelinated neural fibers in nerve bundles that were not in proximity to vessels.

The periosteum of the mouse femur is innervated in part by thinly myelinated Aδ-fibers and peptide-rich unmyelinated sensory C-fibers that express CGRP, with the majority of C-fibers likely transmitting noxious stimuli [27], [28]. Because of the extensive sensory innervation of the periosteum of long bones [4] it has been hypothesized that periosteal distortion is painful [4], [23]. Whether or not tailing is painful to the mouse, as might occur when the coccygeal periosteum, bone, and soft tissues are cut, has been evaluated and depending on the age of the mouse and the specific experimental conditions (including the strain of mouse studied [12]) different conclusions have been drawn. For example, using 4–6 week old C57BL/6 mice, a 2.5 cm amputation of the tip of the tail resulted in long-lasting hyperalgesia [29]. Using younger C57Bl/6 mice (12 and 20 days of age) and a smaller biopsy sample it was concluded that tail amputation had only minor, short-term negative effects on animal welfare [30].

As noted earlier, although we found nerve fibers in the soft tissues of the tail on the day of birth, it was not until PPD 3 that nerves were clearly identified as entering the vertebral perichondrium, and not until PPD 7 that immunostaining revealed distinct neural CGRP reactivity in the perichondrium rather than only near blood vessels. Using microcomputed tomography, Hankenson and colleagues [12] examined the coccygeal vertebrae of 3 day old C57Bl/6 mice and were unable to visualize immature vertebrae (i.e., those having nonmineralized cartilage or only a primary ossification center) in the severed end of a 2 mm tail biopsy. They did, however, visualize immature vertebrae in the severed end of a 5 mm tail biopsy (i.e., a cut made closer to the animal’s body). Correlating the work of Hankenson et al. [12] with the appearance and growth of nerve fibers seen in the current study, it is tempting but nevertheless imprudent to conclude that a 5 mm tail biopsy on PPD 3– with evidence of nerve fibers alongside blood vessels and infiltrating into the vertebral perichondrium – is painful to the animal. This statement is explained further below.

An important corollary question to this study is, “When does the neonatal mouse have sufficient sentience to experience pain if its tail is biopsied?” As stated above, pain cognition is likely to be a function of central and peripheral nervous system maturation. For mice, a species that is moderately neurologically immature at birth, Mellor [31] proposed that they do not exhibit consciousness until at least 4 days of age. This hypothesis was based in part on the work of Diesch et al. [32] who, studying the electroencephalographic responses of rat pups, found pups 5–7 days of age had no electroencephalographic responses to tail clamping and concluded that their pain perception developed gradually from PPD 12 onwards. In contrast, McLaughlin et al. [33] showed that 3 day old rat pups responded to a tonic pain stimulus (formalin injection into the paw) in a manner similar to that of adult rats and suggested that this response may depend on neurosensory and neuromotor maturation rather than upon experimental conditions. Narsinghani and Anand [34], in a detailed review of pain in neonatal rats as young as PPD 0, concluded that neonates do experience pain. More recently, Fitzgerald [35] reviewed experimental evidence in rats and mice that helps support the views of Mellor [31] and Diesch [32]. She noted that a lower percentage of spinal dorsal horn neurons with nociceptive inputs are observed in the first week of life as compared to adult animals and that electrical stimulation of nociceptive C-fibers at that early age failed to evoke a synchronized spike of postsynaptic currents in the dorsal horn of the spinal cord. C-fiber activation occurs as the animal matures. There are large diameter Aδ fibers present at birth; however, Aδ fibers require a longer postnatal period to acquire their full stimulus-response sensitivity, which corresponds to their developing myelination [35]. Additional pain recognition considerations are that neurotransmitter (such as Substance P) release is not consistent in early age neonates and that certain C-fiber synapses within the spinal cord occur as late as PPD 5 [35]. The last finding suggests that “despite the ability of polymodal nociceptors to signal noxious events in the periphery, central nociceptive processing is immature in the postnatal period” [35]. Recently, Davidson and colleagues [36] demonstrated that the spinothalamic tract axons of CD-1 mice reach the brain before birth and exhibit morphological features of functionality while continuing to increase in diameter until PPD 7. Notwithstanding, central nociceptive processing at the level of the brain may remain immature in the neonatal rodent. For example, the expression of Fos protein, an indicator of nociception, did not increase in the brain of PPD 0 rats after an injection of formalin into the paw. It was not until PPD 14 that thalamic labeling was seen in areas generally considered to be involved with the sensation of pain [37].

Our primary research goal was to determine the age at which nerve fibers, and in particular nociceptive fibers, first entered the developing bones of the mouse coccygeal vertebrae. At the level of the proximal biopsy site we found that this occurs in the developing periosteum between PPD 3 and PPD 7. It seems reasonable that the highly innervated developing bone of the coccygeal vertebrae, even when cartilaginous, could contribute to increased pain when a mouse’s tail is injured during the tailing procedure. However, in a neonatal mouse or rat, particularly prior to approximately 12 days of age, a nociceptive stimulus may not result in the conscious perception of pain due to the lack of a competent pain pathway at this age. Using microcomputed tomography on 5 mm tail biopsies from C57BL/6 mice, Hankenson et al. [12] detected immature coccygeal vertebrae at PPD 3 and mature vertebrae at PPD 17 and concluded that tail biopsies for genotyping should preferably be taken at about PPD 14–17. Full neural development in the tail would likely be present at 14–17 days of age but cutting the somewhat less ossified tissue (as compared to after PPD 17) might be less traumatic and subsequently less painful to the animals. Yet, given the potential for true pain perception in rats after PPD 12–14 [32], [37] it would be prudent to consider the use of less invasive alternatives than tailing when collecting tissue for the genotyping of mice. [38]–[40].

## References

[1] Morales ME, Gereau RW (2009) The effects of tail biopsy for genotyping on behavioral responses to nociceptive stimuli. PLoS One doi:10.1371/journal.pone.0006457. 10.1371/journal.pone.0006457 PMC2714470 19649248

[2] Bonaparte (Convenor) D, Cinelli P, Douni E, Hérault Y, Maas A, et al (2013) FELASA guidelines for the refinement of methods for genotyping genetically-modified rodents. A report of the Federation of European Laboratory Animal Science Associations Working Group. Lab Anim 47: 134–145. 23479772 10.1177/0023677212473918

[3] Calvo W, Forteza-Vila J (1969) On the development of bone marrow innervation in new-born rats and studied with silver impregnation and electron microscopy. Am J Anat 126: 355–372. 4188543 10.1002/aja.1001260308

[4] Mach DB, Rogers SD, Sabino MC, Luger NM, Schwei MJ, et al (2002) Origins of skeletal pain: sensory and sympathetic innervation of the mouse femur. Neuroscience 113: 155–156. 12123694 10.1016/s0306-4522(02)00165-3

[5] Hukkanen M, Konttinen YT, Santavirta S, Paavolainen P, Gu XH, et al (1993) Rapid proliferation of calcitonin gene-related peptide-immunoreactive nerves during healing of rat tibial fracture suggests neural involvement in bone growth and remodeling. Neuroscience 54: 969–979. 8341427 10.1016/0306-4522(93)90588-7

[6] Spencer GJ, Hitchcock IS, Genever PG (2004) Emerging neuroskeletal signaling pathways: a review. FEBS Lett 559: 6–12. 14960299 10.1016/S0014-5793(04)00053-5

[7] Gajda M, Litwin JA, Cichocki T, Timmermans J-P, Adriaensen D (2005) Development of sensory innervation in rat tibia: co-localization of CGRP and substance P with growth-associated protein 43 (GAP-43). J Anat 207: 135–144. 16050900 10.1111/j.1469-7580.2005.00434.x PMC1571520

[8] Hunter C, Clegg EJ (1973) The effects of hypoxia on the caudal vertebrae of growing mice and rats. J Anat 116: 227–244. 4783417 PMC1271598

[9] Feik SA, Storey D (1983) Remodelling of bone and bones: growth of normal and transplanted caudal vertebrae. J Anat 136: 1–14. 6339456 PMC1171925

[10] Jerome C, Hoch G (2012) Skeletal System. In: Treuting PM, Dintzis SM, editors. Comparative anatomy and histology: A mouse and human atlas. Amsterdam: Academic Press. 53–70.

[11] Shinohara H (1999) The mouse vertebrae: changes in the morphology of mouse vertebrae exhibit specific patterns over limited numbers of vertebral levels. Okajimas Folia Anat Jpn 76(1): 17–31. 10409842 10.2535/ofaj1936.76.1_17

[12] Hankenson FC, Garzel LM, Fischer DD, Nolan B, Hankenson KD (2008) Evaluation of tail biopsy collection in laboratory nice (*Mus musculus*): vertebral ossification, DNA quantity, and acute behavioral responses. J Am Assoc Lab Anim Sci 47: 10–18. PMC2687139 19049247

[13] Todd ME, Tokito MK (1981) An ultrastructural investigation of developing vasomotor innervation in rat peripheral vessels. Am J Anat 160: 195–212. 6115582 10.1002/aja.1001600206

[14] Karas A, Silverman J (2007) Pain and Distress. In: Silverman J, Suckow MA, Murthy S, editors. The IACUC Handbook, 2^nd^ edition. Boca Raton: CRC Press. 241–286.

[15] Committee for the Update of the Guide for the Care and Use of Laboratory Animals (2011) Guide for the care and use of laboratory animals, 8^th^ edition. Washington, D.C.: The National Academies Press. 220 p.

[16] Henneneberger C, Grantyn R, Rothe T (2000) Rapid genotyping of newborn gene mutant mice. J Neurosci Methods 100: 123–126. 11040374 10.1016/s0165-0270(00)00241-7

[17] Zaidi M, Pazianas M, Shankar V, Bax B, Bax C, et al (1993) Osteoclast function and its control. Exp Physiol 78: 721–739. 8311941 10.1113/expphysiol.1993.sp003721

[18] Naot D, Cornish J (2008) The role of peptides and receptors of the calcitonin family in the regulation of bone metabolism. Bone 43: 813–818. 18687416 10.1016/j.bone.2008.07.003

[19] Patton JT, Kaufman MH (1995) The timing of ossification of the limb bones, and growth rates of various long bones of the fore and hind limbs of the prenatal and early postnatal laboratory mouse. J Anat 186: 175–185. 7649813 PMC1167283

[20] Bjurholm A, Kreicbergs A, Brodin E, Schultzberg M (1988) Substance P- and CGRP-immunorective nerves in bone. Peptides 9: 165–171. 2452430 10.1016/0196-9781(88)90023-x

[21] Ohtori S, Inoue G, Koshi T, Ito T, Yamashita M, et al (2007) Characteristics of sensory dorsal root ganglia neurons innervating the lumbar vertebral body in rats. J Pain 8: 483–488. 17382597 10.1016/j.jpain.2007.01.004

[22] Orita S, Ohtori S, Taniguchi A, Yamashita M, Yamauchi K, et al (2010) Direct evidence for sensory innervation of the dorsal portion of the Co5/6 coccygeal intervertebral disc in rats. Spine 35: 1346–1352. 20354476 10.1097/BRS.0b013e3181c099b0

[23] Martin CD, Jimenez-Andrade JM, Ghilardi JR, Mantyh PW (2007) Organization of a unique net-like meshwork of CGRP+ sensory fibers in the mouse periosteum: implications for the generation and maintenance of bone fracture pain. Neurosci Lett 427: 148–152. 17950532 10.1016/j.neulet.2007.08.055 PMC4444220

[24] Jimenez-Andrade JM, Bloom AP, Mantyh WG, Koewler NJ, Freemen KT, et al (2009) Capsaicin-sensitive sensory nerve fibers contribute to the generation and maintenance of skeletal fracture pain. Neuroscience 162: 1244–1254. 19486928 10.1016/j.neuroscience.2009.05.065 PMC2766854

[25] Koshi T, Ohtori S, Inoue G, Ito T, Yamashita M, et al (2010) Lumbar postereolateral fusion inhibits sensory nerve ingrowth into punctured lumbar intervertebral discs and upregulation of CGRP immunoreactive DRG neuron innervating punctured discs in rats. Eur Spine J 19: 593–600. 20012755 10.1007/s00586-009-1237-9 PMC2899833

[26] Sisask G, Bjurholm A, Ahmed M, Kreicbergs A (1995) Ontogeny of sensory nerves in the developing skeleton. Anat Rec 243: 234–240. 8554179 10.1002/ar.1092430210

[27] Julius D, Basbaum A (2001) Molecular mechanisms of nociception. Nature 413: 203–210. 11557989 10.1038/35093019

[28] Jimenez-Andrade JM, Mantyh W, Bloom AP, Xu H, Ferng AS, et al (2009) A phenotypically restricted set of primary afferent nerve fibers innervate the bone versus skin: Therapeutic opportunity for treating skeletal pain. Bone 46: 306–313 doi:10.1016/j.bone.2009.09.013 19766746 10.1016/j.bone.2009.09.013 PMC2852192

[29] Zhuo M (1998) NMDA receptor-dependent long term hyperalgesia after tail amputation in mice. Eur J Pharma col 349: 211–220. 10.1016/s0014-2999(98)00197-6 9671100

[30] SØrensen DB, Stub C, Jensen HE, Ritskes-Hoitinga M, Hjorth P, et al (2006) The impact of tail tip amputation and ink tattoo on C567BL/6BomTac mice. Lab Anim 41: 19–29. 10.1258/002367707779399383 17234047

[31] Mellor DJ (2010) Galloping colts, fetal feelings, and reassuring regulations: Putting animal-welfare science into practice. J Vet Med Educ 37: 94–100. 20378886 10.3138/jvme.37.1.94

[32] Diesch TJ, Mellor DJ, Johnson CB, Lentle RG (2009) Electroencephalographic responses to tail clamping in anaesthetized rat pups. Lab Anim 43: 224–231. 19237459 10.1258/la.2008.0080083

[33] McLaughlin CR, Lichtman AH, Fanselow MS, Cramer CP (1990) Tonic nociception in neonatal rats. Pharmacol Biochem Be 36: 859–862. 10.1016/0091-3057(90)90090-5 2217514

[34] Narsinghani U, Anand KJS (2000) Developmental neurobiology of pain in neonatal rats. Lab Anim (NY) 29(9): 27–39. 10.1038/5000089 11381240

[35] Fitzgerald M (2005) The development of nociceptive circuits. Nat Rev Neuroscience 6: 507–520. 15995722 10.1038/nrn1701

[36] Davidson S, Truong H, Giesler Jr G (2010) A quantitative analysis of spinothalamic tract neurons in adult and developing mouse. J Comp Neurol 518: 3193–3204. 20575056 10.1002/cne.22392 PMC2996724

[37] Barr GA (2011) Formalin-induced c-fos expression in the brain of infant rats. J Pain 12: 263–271. 21146467 10.1016/j.jpain.2010.09.005 PMC3062261

[38] Irwin MH, Moffatt RJ, Pinkert CA (1996) Identification of transgenic mice by PCR analysis of saliva. Nat Biotechnol 14: 1146–1148. 9631068 10.1038/nbt0996-1146

[39] Schmitteckert EM, Prokop CM, Hedrich HJ (1999) DNA detection in hair of transgenic mice – a simple technique minimizing the distress on the animals. Lab Anim 33: 385–380. 10778788 10.1258/002367799780487922

[40] Meldgaard M, Bollen PJ, Finsen B (2004) Noninvasive method for sampling and extraction of mouse DNA for PCR. Lab Anim 38: 413–417. 15479556 10.1258/0023677041958981

